# A simple strategy based on fibers coated with surfactant-functionalized multiwalled carbon nanotubes to improve the properties of solid-phase microextraction of phenols in aqueous solution

**DOI:** 10.1186/s13065-020-00665-7

**Published:** 2020-02-19

**Authors:** Xueqing Zhou, Yanli Xie, Zhendong Zhao, Wenyan Fu

**Affiliations:** 1 Analytical and Testing Center, Hainan University, Haikou, 570228 China; 2 College of Materials and Chemical Engineering, Hainan University, Haikou, 570228 China

**Keywords:** Functionalized multiwalled carbon nanotube, Solid-phase microextraction, Surfactant, Phenol

## Abstract

**Methods and experiments:**

In this study, a functionalized multiwalled carbon nanotube (MWCNT)-coated solid-phase microextraction (SPME) fiber was developed for concentrating analytes in aqueous samples. Sodium deoxycholate (NaDC) was used as a dispersing agent for non-covalent modification of MWCNTs. The coating showed porous structure and large adsorption capacity. To investigate the capability of this MWCNTs/NaDC SPME fiber, it was applied to the analysis of phenols in aqueous solution. After extraction, the analytes were desorbed in an acetonitrile–water solution and analyzed using high-performance liquid chromatography.

**Results:**

The MWCNTs/NaDC fiber exhibited good analytical performance, and fine preparation reproducibility was obtained with the relative standard deviations (RSDs) ranging from 4.9% to 10.2% (n = 6) in one batch, from 5.7% to 11.9% (n = 3) among different batches. Under the optimum extraction conditions, the detection limits were 0.15–0.30 ng/mL(S/N = 3), the linear detection ranges were 1–100 ng/mL (R^2^ ≥ 0.9997) for these analytes, and good recoveries (80.3–95.4%) were obtained for the spiked samples.

**Conclusion:**

This is a simple and accurate pretreatment method for the analysis of phenols in aqueous samples.

## Introduction

Phenols are hydroxyl-containing derivatives of aromatic hydrocarbons, which are one of very toxic organic contaminants [1, 2]. Due to the dense population and industry, the phenols pollution in environment is becoming increasingly serious [3]. Monitoring of phenols contaminants is particularly important, which is the basis and prerequisite for the control and remediation of phenols. Some conventional methods, such as Liquid–liquid extraction and solid-phase extraction, were often used to extract analytes in aqueous samples. However, these techniques were usually time-consuming and required toxic organic solvents [4, 5]. Therefore, ideal sample-preparation techniques are commonly required.

In the past few years, solid-phase microextraction (SPME) have been developed for the extraction of phenols [6–9] The obvious advantages of SPME are solvent-free process, simplicity of operation, and a short extraction time, which reduces contamination of the sample and loss of analytes [10, 11]. Besides, it can combine sampling, extraction and enrichment into a single step [12]. In these methods, commercial non-polar polydimethylsiloxane (PDMS) coated SPME fibers are widely used for extraction of phenols in environmental water samples [13, 14]. For instance, Quintana et al. [15] and Montero et al. [16] applied PDMS coated SPME to extract phenols from environmental samples. Yu et al. [17] selected commercial PDMS/DVB fiber for the determination of phenols and related chlorophenols in water. However, due to the polarity of phenols, the application of PDMS fibers has to be accompanied with derivatization, which increases the triviality of pretreatment. And their performance is not always satisfactory for the extraction of large numbers of varied analytes due to their thermal instability or limited selectivity [18]. To obtain high extraction efficiencies for these compounds, various types of SPME coatings have been investigated, including molecularly imprinted polymers [19], ionic liquids [20], metal–organic frameworks [21], and carbon materials [22–25].

Multiwalled carbon nanotubes (MWCNTs) have unique electronic, mechanical, and chemical properties and have attracted attention in recent years [26–30] MWCNTs contain internal cavities that are large enough to allow analytes to penetrate, and their surfaces and interspaces within nanotube bundles provide excellent sorption [31]. Because of their unique properties and their hydrophobic character, MWCNTs are superior adsorbents for aliphatic hydrocarbons [32], polycyclic aromatic hydrocarbons (PAHs) [33], phthalates [34], and volatile organic compounds [35, 36]. Jiang et al. [37] first employed CNTs as SPE adsorbent for the determination of bisphenol A, 4-nonylphenol and 4-tert-octylphenol, which showed good performance. In addition, functionalization of the CNTs plays a key role in selectivity for polar compounds. Hu et al. [38] synthesized amino modified multi-walled carbon nanotubes/polydimethylsiloxane (MWCNTs-DDM/PDMS) coating for stir bar sorptive extraction, which was successfully applied to the analysis of phenols in environmental water and soil samples. Ai et al. [39] reported an ionic liquid functionalized multiwalled carbon nanotubes–polyaniline (MWCNT@IL/PANI) nanocomposite coating. This coating exhibited high extraction efficiency.

Moreover, it was reported that surfactants or macromolecules could interact strongly with MWCNTs, which have an effect on their structures and polarity, and modification of MWCNTs with surfactants or macromolecules can increase their solubility and dispersibility [40, 41]. It can be speculated that surfactants modified MWCNTs will be an efficient SPME coating material for the extraction of phenols because of the possible interaction between surfactants modified MWCNTs and the target phenols including hydrophobic interaction and intermolecular hydrogen bond.

In this study, a new sodium deoxycholate functionalized multiwalled carbon nanotube-coated (MWCNTs/NaDC) fiber was fabricated, and was used to analysis of phenols from environmental samples. The combination of SPME with high-performance liquid chromatography (HPLC) provides an accurate and sensitive method for the determination of phenols in aqueous solution, and was applied to seawater samples from the South China Sea and Wastewater.

## Experimental

### Reagents and materials

Phenol, *p*-nitrophenol (4-NP), *o*-nitrophenol (2-NP), 2, 4-dimethylphenol (2,4-DMP), and 2,4-dichlorophenol (2,4-DCP) standards were purchased from Sinopharm Chemical Reagent Co., Ltd. (Shanghai, China). Hydrogen nitrate (AR grade, purity 65–68%) and sulfuric acid were purchased from Guangzhou Chemical Reagents (Guangzhou, China). HPLC-grade acetonitrile (ACN), methanol, isopropanol, and formic acid were from Thermo Fisher Scientific Co. (Waltham, MA). Fused-silica fibers (120 µm i.d.) were obtained from Ruifeng Chromatographic Device Co. Ltd (Yongnian, China). MWCNTs (20–30 nm, purity > 98% mass fraction) were purchased from Sigma–Aldrich (St. Louis, MO). SPME hand shank and PDMS/DVB fiber (1 cm length, 65 µm thick, Supelco, USA) were purchased from Sigma-Aldrich Co., Ltd. (Shanghai, China). Acrylic ester was obtained from Guangzhou Chemical Reagents. Sodium deoxycholate (NaDC) was purchased from Aladdin Chemistry (Shanghai, China). Ultrapure water was prepared with a Milli-Q water purification system (Millipore, Bedford, MA). All other chemicals were of analytical grade.

### Instruments and conditions

Sample analyses were carried out using a Waters (Milford, MA) e2695 system equipped with a 2998 photodiode array detector. A C18 column (250 mm × 4.6 mm i.d., 5 µm particle size, Agela) was used for the chromatographic separation. The mobile phase was a mixture of 0.2% acetic acid (A) and acetonitrile (B) at a flow rate of 1.0 mL/min. We used the following gradient elution: 0–5 min, A = 70% and B = 30%; 20 min, A = 20% and B = 80%; 21 min, A = 10% and B = 90%; 25 min, A = 10% and B = 90%; then the ratio of solvent B decreased to 30% in 2 min and kept for 3 min to equilibrate the column. The total run time was about 25 min. The column temperature was 30 °C and the wavelength was 280 nm.

Transmission electron microscope (TEM) images were recorded using a JEM 2100 instrument (JOEL, Tokyo, Japan). SEM observation was executed using a Hitachi S-3000 N(Japan) scanning electron microscope after fixing the samples on a brass holder and coating them with gold. Fourier transform infrared (FT-IR) spectra of the materials were obtained on a TENSOR 27 spectrometer (Bruker, Ettlingen, Germany). The freeze-dried samples were mixed with KBr compressed into semitransparent KBr pellets before the measurement. XRD spectra were recorded on a D8 Advance X-ray diffractometer ((Bruker, Germany) with Cu-K a radiation (λ = 0.154 nm). The XRD was operated at 40 kV and 40 mA in a step scan mode. The scanning speed was 0.025°/s. XRD measurements were performed over a 2θ range of 10°–40°.

### Synthesis of functionalized MWCNTs

**Fig. 1:**
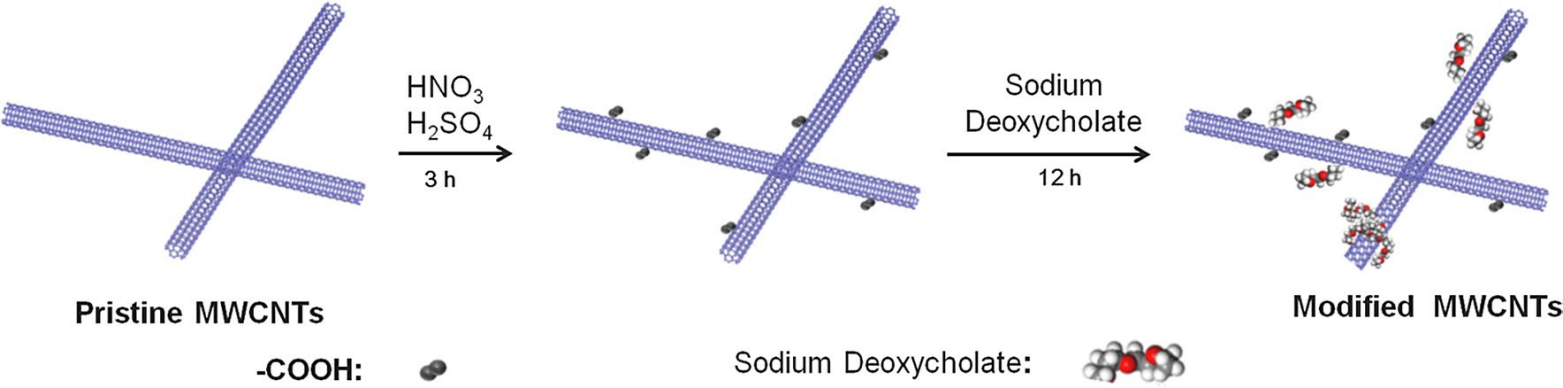
Synthesis of the MWCNTs/NaDC

Functionalized MWCNTs (MWCNTs/NaDC) were synthesized as follows. First, 2 g of pristine MWCNT powder was added to 100 mL of a mixture of HNO_3_ and H_2_SO_4_ (1:3, v/v). The mixture was then heated in a water bath at 80 °C for 3 h with stirring. The processed MWCNTs were collected by filtration and washed with deionized water until the pH approached neutral, and then dried at 60 °C for further use. Second, the processed MWCNTs were modified by sodium deoxycholic acid (NaDC) with a mass ratio of 1:1 (see Additional file 1). The suspension was ultrasonic for 30 min in the ultrasonic crushing instrument, and then by magnetic stirring for 12 h. The resulting solution was filtered, washed with ultrapure water, and dried at 60 °C for 24 h. Figure 1 presents the synthetic strategy for the MWCNTs/NaDC.

### Preparation of the MWCNTs/NaDC SPME fiber

The MWCNTs**/**NaDC SPME fiber was fabricated using the following processes. First, a silica fiber was cut to 10 cm. Then, one end (2.0 cm) was burned on an alcohol blast burner to remove the protecting polyimide layer. Subsequently, the fiber was washed thoroughly with 10 mL of water and 10 mL of acetonitrile in sequence, and dried at 60 °C for 12 h. Next, the fiber was parallelly dipped in epoxy glue for 30 s, and then coated. The unnecessary epoxy glue was removed with a tweezers to generate a homogeneous film of glue. And the equal in quality of MWCNT/NaDC was immobilized on every silica fiber by silicone glue. The coated section was 2.0 cm long. Finally, the proposed SPME fiber was heated to 200 °C for 5 h to remove any contaminants.

For comparison, the preparation procedure for MWCNTs fiber was the same as that for the MWCNTs/NaDC-coated SPME fiber as described above.

### SPME procedure

For phenol analysis, all extraction experiments were carried out in a 10-mL working solution, which was introduced to a 20-mL amber vial capped with a polytetrafluoroethylene-coated septum. The solution was agitated at 1100 rpm by magnetic stirring with a Teflon-coated stir bar. To perform the extraction, the MWCNTs/NaDC-coated SPME fiber was immersed in a water sample for a certain time. After extraction, the fiber was removed and placed in a 5-mL vial. Then, the analytes were desorbed in a 2 mL solution of 70% ACN solvent, and 10 µL of the stripping solvent was used for HPLC analysis. The chromatographic peak area was used to evaluate the extraction efficiency under different conditions. The MWCNTs/NaDC SPME fiber could be reused after rinsing with the 70% ACN solvent. To avoid memory effects, the SPME fibers were desorbed in a 70% ACN solvent after extraction until the desorption solution had a flat baseline on injection into the HPLC.

### Analysis of real samples

Water samples were collected from the South China Sea near Baishamen, Yangpu, and Holiday Beach. Wastewater sample were collected from a chemical plant. These samples were analyzed immediately after sampling without any pretreatment.

### Repeatability tests

All experiments including the optimization of extraction conditions and the real samples analysis were performed in triplicate. The chromatographic peak area was used for quantification, and the experimental results were expressed as mean ± standard deviation.

## Results and discussion

### Preparation and characterization of modified MWCNTs

**Fig. 2:**
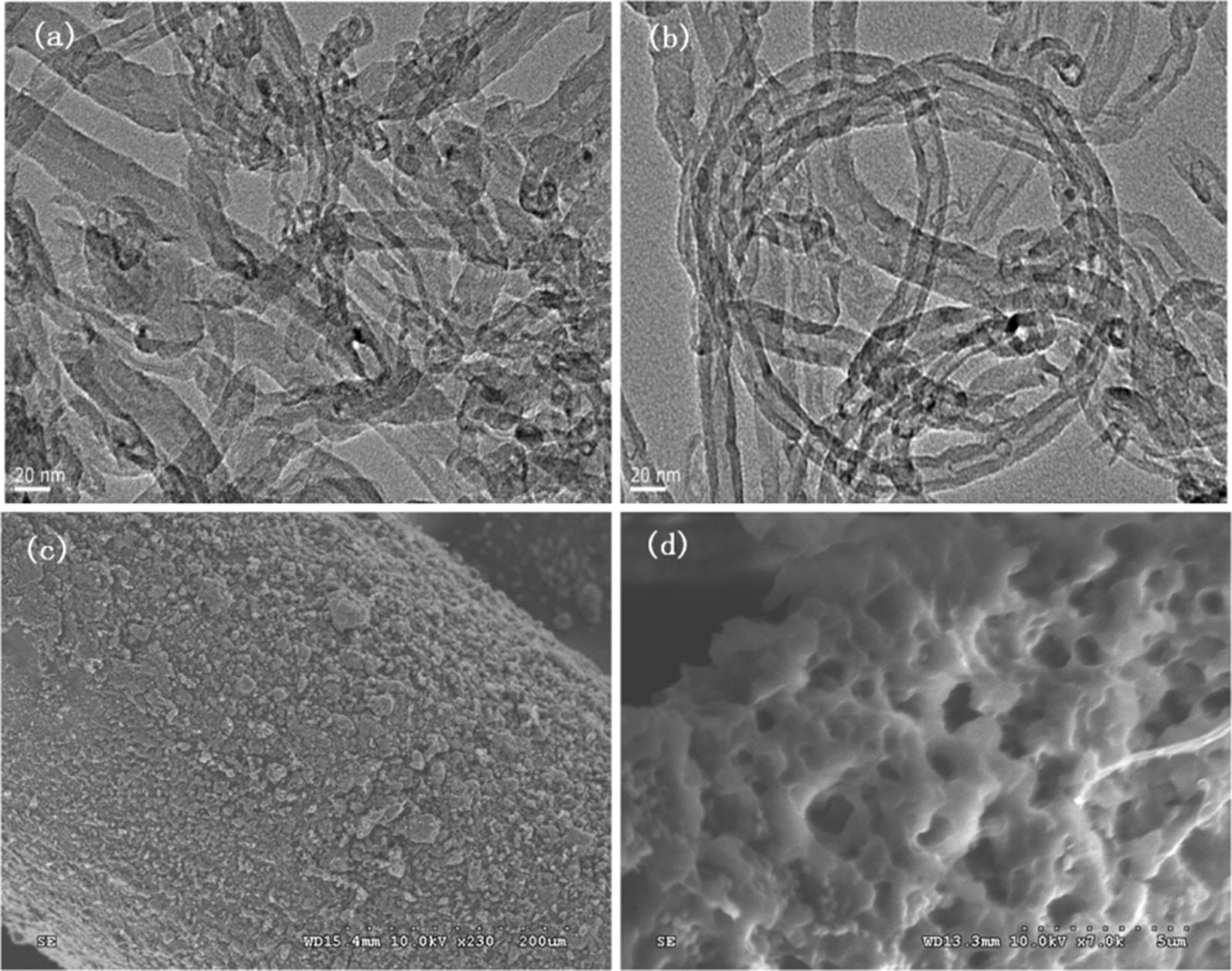
TEM image of the original MWCNTs (**a**), TEM image of the modified MWCNTs after processing with NaDC (**b**), and SEM image of the MWCNTs/NaDC coating surface of the silica fiber (**c**, **d**)

The TEM and SEM images of the coating are shown in Fig. 2. As can be seen, the TEM image of the original MWCNTs (Fig. 2a) showed an aggregated structure, whereas the MWCNTs/NaDC (Fig. 2b) was well-dispersed in coating. SEM image reveal the presence of MWCNTs/NaDC coating the surface of the silica fiber in Fig. 2c. The coating presents net-like and porous structure, and the coating thickness was approximately 20 µm (Fig. 2d). The MWCNTs/NaDC fiber had high specific surface area, which was favorable for the adsorption/extraction of analytes. So it could be concluded that the introduction of NaDC could avoid the aggregation of MWCNTs and improve the dispersion of MWCNTs on SPME fiber.

**Fig. 3:**
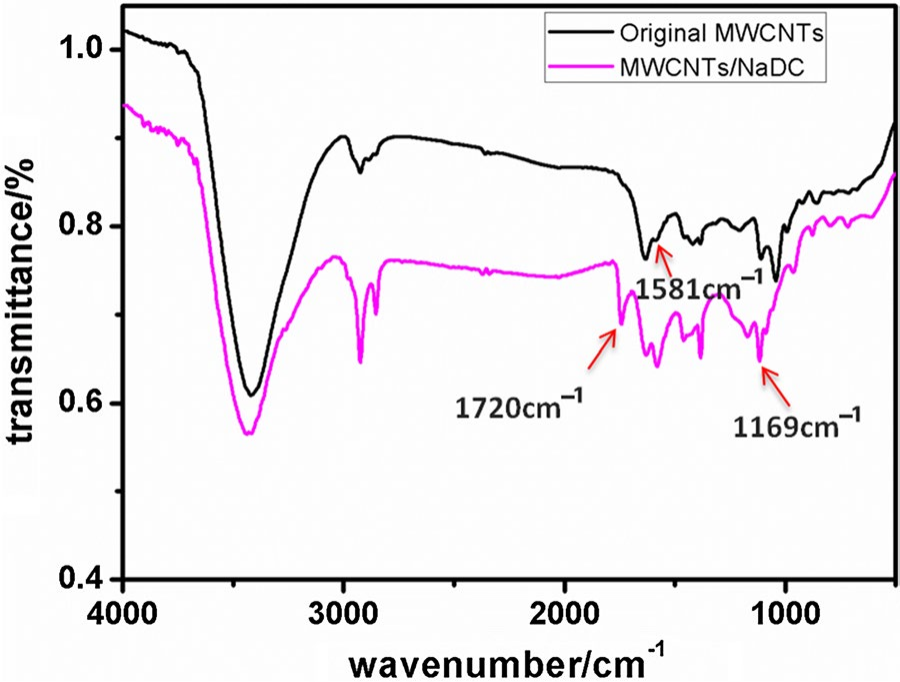
The FT-IR spectrum of the MWCNTs/NaDC and original MWCNTs

The FT-IR spectra of MWCNTs/NaDC and original MWCNTs are shown in Fig. 3. The absorption peak at ~ 1720 cm^−1^ was the characteristic peaks of stretching vibration of the C = O in the spectral curve of MWCNTs/NaDC. The absorbance peaks at approximately 1169 cm^−1^assigned to the C–OH stretch. After NaDC modification, because of the interaction between NaDC with MWCNTs, the stretching vibration of C = C (~ 1581 cm^−1^) peak move higher wavenumber [42]. The result showed that NaDC is mainly combined with the surface of MWCNTs–COOH by random adsorption in a non-covalent modification way.

### Enrichment factor of SPME

For the equilibrium adsorption experiments, the enrichment factor of SPME depended on the interactions (i.e., π–π, hydrogen bonding, and electrostatic) between the analytes and MWCNTs/NaDC. The enrichment factor (EF) is defined as the ratio of the extracted ananlyte with regard to the initial amount. And the chromatographic peak area was used for quantification of the final analyte concentration after extraction, which was obtained by direct injection of 10 µL of standard solution.

The enrichment factor (*EF*) was calculated as follows:

1 $$ \varvec{EF} = \frac{{\varvec{C}_{\varvec{A}} }}{{\varvec{C}_{\varvec{i}} }} $$

where *C*_A_ was the amount of analyte extracted by SPME, *C*_i_ is the initial concentration.

The SPME using the MWCNTs/NaDC fiber was carried out with phenols concentration of 10.0 ng/mL. The results are represented in Table 1. As can be seen, the MWCNTs/NaDC fiber presents high EF values for five phenols. It has been reported that the *van der Waals force*, H-bonding interactions from solutes as hydrogen-bonding donors, and followed by π-electron polarizability, may play important roles on the adsorption of phenols by MWCNTs in the aqueous environment [43].

**Table 1:** Physico-chemical properties of five phenols and the enrichment factor (*EF*) obtained with the MWCNTs/NaDC fiber

Compound	Structure	Molecular weight	*C*_*A*_ (ng/mL)^a^	*EF*^b^
Phenol		94	9.12	91.2 ± 0.5
4-NP		139	8.96	89.6 ± 0.4
2-NP		139	8.53	85.3 ± 0.3
2,4-DMP		122	8.14	81.4 ± 0.2
2,4-DCP		163	9.01	90.1 ± 0.3

Samples of South China seawater (10 mL) were spiked with phenols at 10.0 ng/mL and extracted

^a^*C*_*A*_ was calculated as the concentration of the analyte extracted

^b^The enrichment factor (*EF*) of the analytes was calculated as the ratio of the analyte concentration after extraction to that in the original sample

### Effect of experimental conditions on the extraction and desorption with the MWCNTs/NaDC SPME fiber

To evaluate the performance of the MWCNTs/NaDC SPME fiber, different experimental parameters that could affect extraction efficiency, including the extraction time, the inorganic salt concentration, the composition of the elution solvent, and desorption time, were investigated and optimized.

#### Extraction time

**Fig. 4:**
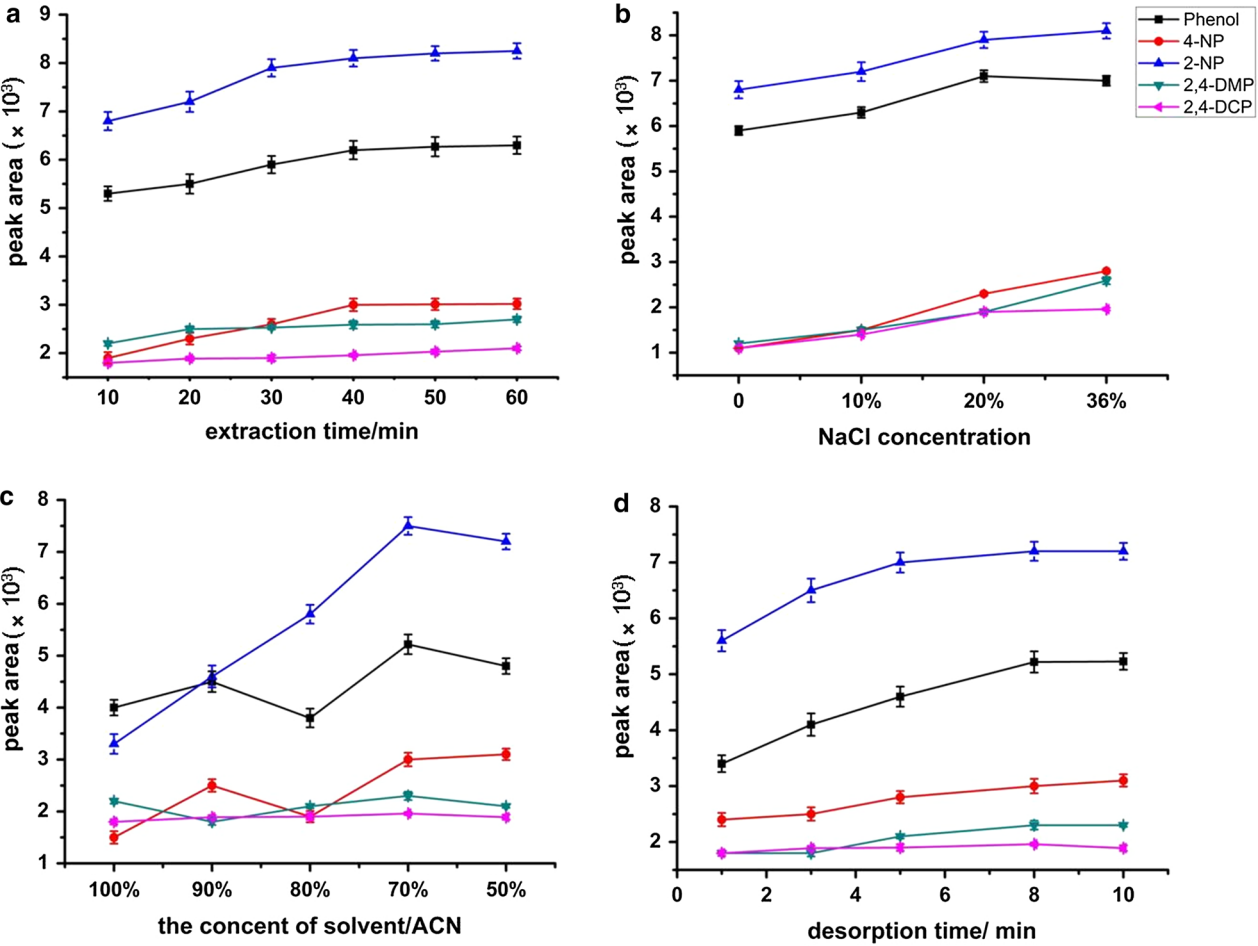
Effects of the experimental conditions on the extraction efficiency of the MWCNTs/NaDC SPME fiber for 100 ng/mL each analyte. **a** Extraction time, **b** ionic strength, **c** the composition of the elution solvent, and** d** desorption time. Errors bars show the standard deviation of the mean (*n* = 3)

Extraction time is an important factor that influences the extraction efficiency, and there is a correlation between the extraction amount and the extraction time. The effect of extraction time on the extraction efficiency of the MWCNTs/NaDC coating was studied by varying the extraction time from 10 min to 60 min. Extraction profiles for the five phenols are shown in Fig. 4a. The results indicated that the extraction efficiency increased with the exposure time, and the extraction equilibrium was reached in 20 min for 2,4-DCP, 30 min for 2,4-DMP, 40 min for phenol and 4-NP, and 50 min for 2-NP. All phenols reached extraction equilibrium within 50 min. Therefore, 50 min was chosen as the preferred extraction time.

#### Ionic strength

Ionic strength is another parameter influencing the extraction efficiency of analytes in an aqueous solution. Addition of inorganic salt can decrease the solubilities of analytes in an aqueous sample and enhance their concentrations in the adsorbent. Therefore, the influence of ionic strength was investigated by adding different proportions of NaCl (0–36% w/v) as a salting-out agent (Fig. 4b). The results showed that most compounds had higher extraction efficiencies with high salt concentrations than with low salt concentrations, and 36% (w/v) NaCl was chosen for subsequent extractions.

#### Composition of the elution solvent

The effect of the composition of the elution solvent on desorption was also investigated (Fig. 4c). A mixture of ACN and water gave good solubility for the phenols on the modified MWCNTs fiber. Generally, as the water volume fraction increased, the amount of desorption also increased for most phenols. The optimum desorption was achieved with an ACN volume fraction of 70%. Consequently, 70% ACN was used in subsequent experiments.

#### Desorption time

The effect of desorption time was investigated with ultrasonication for between 1 and 10 min (Fig. 4d). All the phenols were desorbed almost completely within 8 min. Increasing the time above 8 min did not considerably increase the desorption efficiency. Therefore, 8 min was chosen as the optimum desorption time.

The optimum conditions for SPME of phenols were an extraction time of 50 min, NaCl content of 36% (w/v), ACN volume fraction of 70%, and desorption time of 8 min.

### Comparison of the MWCNTs/NaDC fiber with a commercial PDMS/DVB fiber

**Fig. 5:**
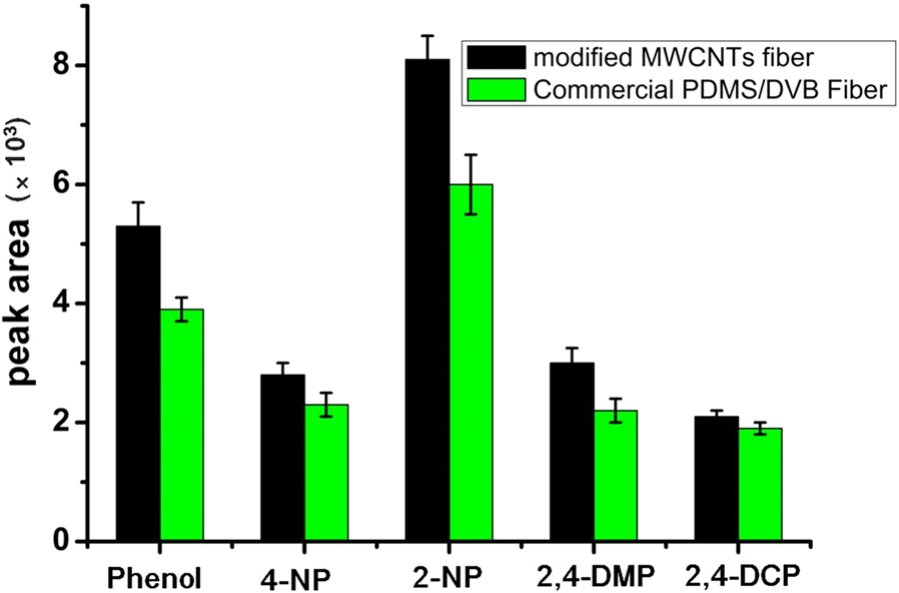
Peak areas obtained with the MWCNTs/NaDC fiber and commercial PDMS/DVB fiber for phenols. The extraction of MWCNTs/NaDC fiber and commercial PDMS/DVB fibers for phenols were under the optimized conditions. Extraction time: 50 min; stirring speed: 1100 rpm; NaCl concentration: 36% (w/v); concentration of phenols: 100.0 ng/mL for Phenol, 4-NP, 2-NP, 2,4-DMP and 2,4-DCP. Error bars show the standard deviation of the mean (*n* = 3)

The extraction performance of the MWCNTs/NaDC fiber was compared with a commercial PDMS/DVB fiber (1 cm length, 65 µm thick), which was suitable for the SPME of polar and half volatile compounds such as amines, phenols and parabens, etc. [44]. The SPME using the MWCNTs/NaDC and PDMS/DVB fiber was carried out with a phenol concentration of 100.0 ng/mL. The results are represented in Fig. 5. The relative response of the MWCNTs/NaDC fiber was higher than that of the commercial PDMS/DVB fiber for phenols. The high selectivity of the coating for phenols can be attributed to the hydrogen interactions with the analytes and the large surface area of the MWCNTs, which facilitated the phenols adsorption.

### Comparison of MWCNTs/NaDC fiber with MWCNTs fiber

**Fig. 6:**
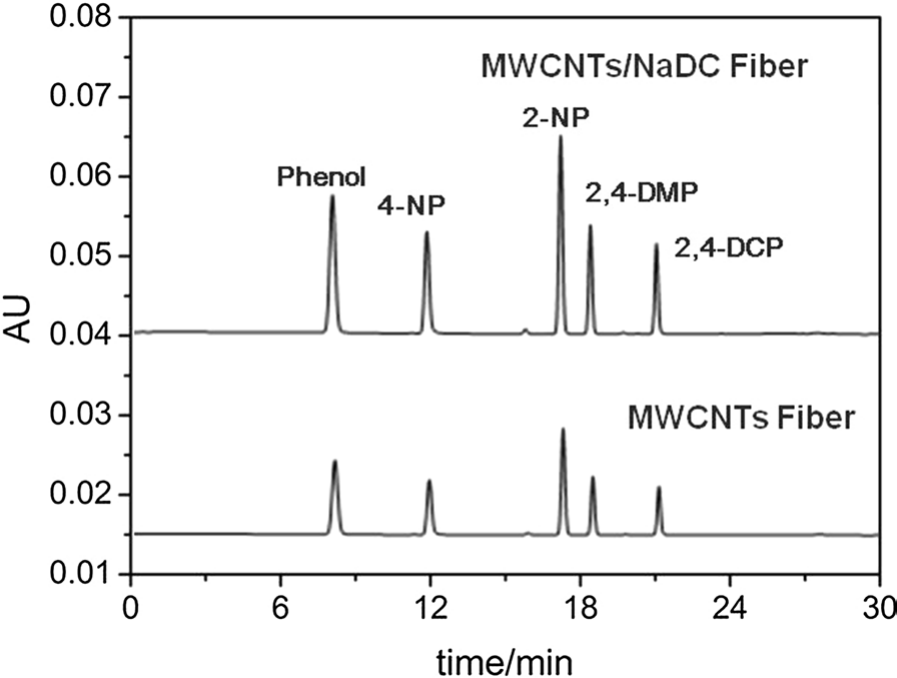
Chromatograms of the phenols standard solution containing 100 ng/mL obtained with the MWCNTs/NaDC and the pristine MWCNTs fibers

To evaluate the usefulness of the MWCNTs/NaDC Fiber, the adsorption efficiency of MWCNTs/NaDC with pristine MWCNTs were compared. The SPME analysis of the aqueous sample was executed by testing the sample spiked with phenols at 100.0 ng/mL. As presented in Fig. 6, the peak area of the fiber with MWCNTs/NaDC increased additionally as compared to the pristine MWCNTs fiber, so that the better performance of the MWCNTs/NaDC fiber was achieved. This can be explained by the fact that the modification of MWCNTs with NaDC increased the conjugation interaction between the coating and the target phenols. And the performance of MWCNT-coated SPME fiber can be enhanced by the complete dispersion and proper assembly of the nanotubes [45].

### The stability of MWCNTs/NaDC coating

**Fig. 7:**
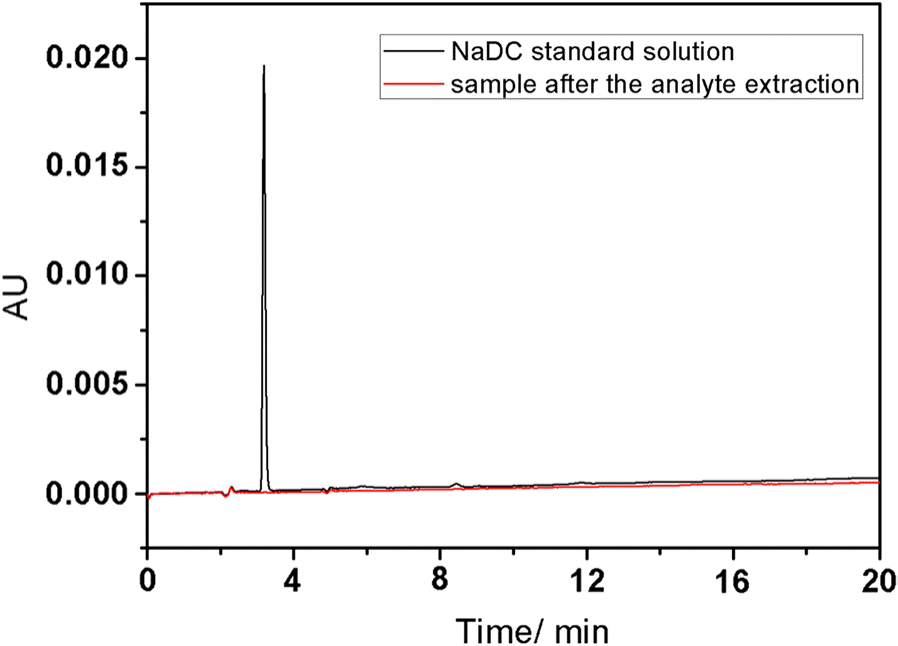
Chromatograms of NaDC standard solution and sample after the analyte extraction.(Agela C18 column(250 mm × 4.6 mm i.d); The mobile phase was methyl alcohol: water (3:7) at a flow rate of 1.0 mL/min; The column temperature was 30 °C and the wavelength was 210 nm)

The stability of MWCNTs/NaDC coating is very important for practical applications. To check the stability of the extractive phase, the residual amount of NaDC in aqueous solution after the analyte extraction was determined. Figure 7 showed the chromatograms of NaDC standard solution and sample after the analyte extraction, almost no NaDC was found in aqueous solution, which demonstrates that the stability of MWCNTs/NaDC coating was good, no leaching of NaDC from the fiber to the water sample appeared.

### The lifetime of the coating

**Fig. 8:**
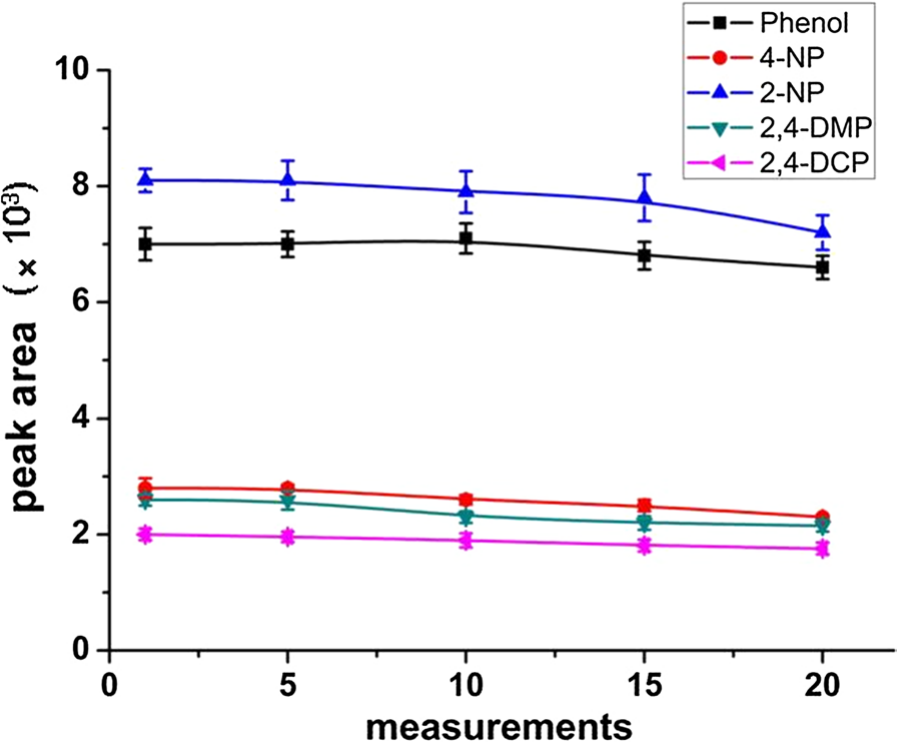
Peak areas of the phenols as the function of the number of measurements performed with a single MWCNTs/NaDC fiber

The lifetime of a coating is also very important for practical applications. The reproducibility of the MWCNTs/NaDC fiber extraction was investigated. After repeating phenol extraction and elution 20 times, the peak areas in the SPME chromatograms did not change remarkably (Fig. 8). These results demonstrated that there was no apparent loss in performance of MWCNTs/NaDC fiber after 20 times of extraction and desorption cycles, indicating that the coating is quite stable and reproducible.

### Method validation and application to real samples

Under the optimized conditions, calibration curves were constructed for phenols analysis (Table 2). The linear ranges for all tested analytes were 1–100 ng/mL with good correlation coefficients (*R*^2^ > 0.9997). The limits of detection (LODs) were calculated at a signal-to-noise ratio of three, and the LOD ranges were 0.15–0.30 ng/mL. The LOQ were estimated from the S/Ns of 10, and LOQ values were in the range of 0.60–1.20 ng/mL. The results showed that the RSD range between 4.9 and 10.2% from single fiber, and range between 5.7 and 11.9% from fiber to fiber with three replicates.

**Table 2:** Analytical performance for HPLC determination of phenols using the MWCNTs/NaDC fiber

Compound	Linear equation	*R*^2^	LOD (ng/mL)	LOQ (ng/mL)	RSD (%, *n* = 6)^a^	RSD (%, *n* = 3)^b^
Phenol	*y* = 54409*x* + 1438.9	0.9997	0.16	0.64	4.9	7.6
4-NP	*y* = 32887*x* + 368.02	0.9997	0.28	1.12	5.1	11.9
2-NP	*y* = 52340*x* + 711.27	0.9998	0.15	0.60	6.3	8.8
2,4-DMP	*y* = 26372*x* + 775.15	0.9998	0.30	1.20	10.2	9.6
2,4-DCP	*y* = 21672*x* + 224.74	0.9998	0.29	1.16	7.1	5.7

^a^Single fiber with six replicates

^b^Fiber to fiber with three replicates. The Linear ranges were 1–100 ng/mL for five phonels

The comparison of the proposed method with other methods was summarized in Table 3, which including SPME, Stir bar sorptive extraction (SBSE), Stir bar-supported micro-solid-phase extraction (SB-µ-SPE) and et al. The LODs of the developed method were lower than that obtained by MWCNTs-COOH fiber SPME [6] and Stir bar-supported micro-solid-phase extraction [47], and comparable with that achieved by MWCNTs-DDM/PDMS SBME [38], PDMS-SBSE [16] and SB-µ-SPE [46]. Although the LODs of this method were higher than that obtained by PDMS-SBSE [16], the RSD of this method were lower. In short, our method is comparable to existing methods and allows for trace analysis in real samples.

**Table 3:** Comparison of the proposed method with other methods

Method	Detection system	LOD (ng/mL)	RSD (%)	Liner range (ng/mL)	References
SPME^a^	HPLC–UV	0.25–3.67	4.25–12.95	10.8–1585	[6]
SPME^b^	GC-MSMS	0.26–2.63	2.08–9.02	1–1000	[21]
MWCNTs-DDM/PDMS SBSE^c^	HPLC–UV	0.14–1.76	6.2–11.6	1–1000	[38]
PDMS-SBSE^d^	GC–MS	0.1–0.4	6–27	1–15	[16]
SB-µ-SPE^e^	GC–MS	0.24	4.50	1–600	[46]
SUPRAS-microextraction^f^	HPLC–DAD	1–4	4.70–7.27	10–150	[47]
MWCNTs/NaDC SPME	HPLC–UV	0.15–0.30	5.7–11.9	1–100	Present study

^a^MWCNTs-COOH fiber SPME

^b^Carboxylated solid carbon spheres SPME

^c^Amino modified multi-walled carbon nanotubes/polydimethylsiloxane coated stir bar sorptive extraction

^d^Polydimethyl siloxane (PDMS) stir bar sorptive extraction

^e^Stir bar-supported micro-solid-phase extraction

^f^Supramolecular solvent based microextraction

**Fig. 9:**
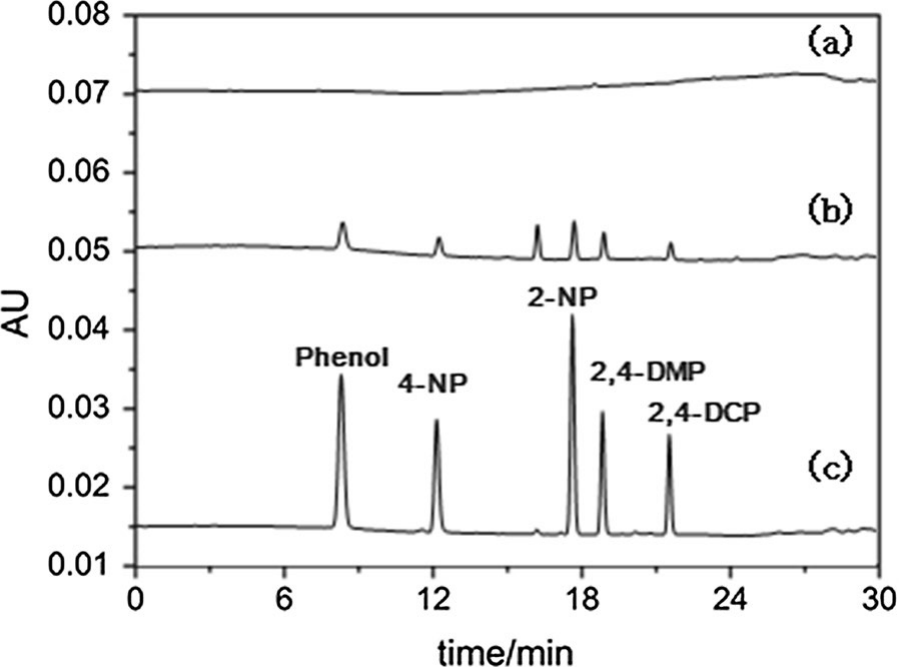
Chromatograms of phenols extracted by the MWCNTs/NaDC fiber from (a) a blank Yangpu seawater sample and (b) the same seawater sample spiked with a phenols standard solution at 10.0 ng/mL. (c) The 100.0 ng/mL phenols standard solution used for spiking

Then, the optimized method was applied to the determination of phenols in samples from the South China Sea and Wastewater. 10 mL of aqueous solution was extracted by this novel MWCNTs/NaDC fiber without any pretreatment, the results indicated that Phenol was detected in the wastewater(3.64 ng/mL), while the concentrations of 2-NP, 4-NP, 2,4-DMP and 2,4-DCP were below the LODs. No phenolic compounds were found in South China Seawater. Spiking experiments were then performed to evaluate the accuracy of the established method, and the samples were spiked with phenols at 10.0 and 100.0 ng/mL. Figure 9 showed the chromatograms of phenols extracted by the MWCNTs/NaDC fiber from real water sample and spiked samples. As listed in Table 4, the recovery ranges were 85.6–93.1% (relative standard deviation < 7%) for seawater samples from the Yangpu area, 88.6–95.4% for samples from the Baishamen area, 80.3–87.3% for samples from the Holiday Beach area, and 82.6–90.5% for wastewater samples. The precision and accuracy of the present method were acceptable.

**Table 4:** Analytical results for the determination of phenols in seawater samples from the South China Sea and Wastewater

Samples	Phenol	4-NP	2-NP	2,4-DMP	2,4-DCP
Yangpu					
Concentration (ng/mL)	nd^c^	nd	nd	nd	nd
Recovery^a^	93.1	85.6	92.6	89.3	91.2
RSD (%, *n* = 3)	4.2	5.0	6.8	5.1	4.9
Baishamen					
Concentration (ng/mL)	nd	nd	nd	nd	nd
Recovery^b^	95.1	89.6	94.2	88.6	95.4
RSD (%, *n* = 3)	6.4	5.9	6.8	7.1	8.2
Holiday beach					
Concentration (ng/mL)	nd	nd	nd	nd	nd
Recovery^b^	81.6	85.4	87.3	81.9	80.3
RSD (%, *n* = 3)	5.9	6.8	7.1	6.9	8.3
Wastewater					
Concentration (ng/mL)	3.64 ± 0.17	nd	nd	nd	nd
Recovery^b^	85.3	84.1	85.6	90.5	82.6
RSD (%, *n* = 3)	5.9	6.8	7.1	6.9	8.3

^a^Spiked with phenols at 10.0 ng/mL

^b^Spiked with phenols at 100.0 ng/mL

^c^nd = not detected. Three parallel experiments were conducted for each sample

## Conclusions

In this work, a new MWCNTs/NaDC SPME fiber coupled with HPLC for trace analysis of phenols has been developed. The MWCNTs/NaDC fiber exhibited high extraction efficiencies, a wide linear range, low LODs and satisfactory reproducibility for phenols. The high adsorption capacity can be attributed to hydrogen bonding between the hydroxyl and carboxyl groups of the MWCNTs/NaDC surface and phenols. The mutual effect between the MWCNTs and sodium deoxycholate changed the electrical and steric resistance of the MWCNTs, and improved their solubility and dispersibility. The proposed fiber has better detection sensitivity than commercial PDMS/DVB fibers. The LOD range of the proposed method for phenols analysis was 0.15–0.30 ng/mL. These results broaden the potential for application of MWCNTs in the analysis of trace compounds in aqueous samples.

## Supplementary information

**Additional file 1.** Additional tables.

## Data Availability

All data generated or analysed during this study are included in this published article and its additional files.

## References

[1] The list of priority substances in the field of water policy and amending directive, Council directive 2455/2001/ECC, Official Journal L331, November 20 (2001) 1

[2] Busca G, Berardinelli S, Resini C, Arrighi L (2008). Technologies for the removal of phenol from fluid streams: a short review of recent developments. J Hazard Mater.

[3] UNICEF and World Health Organization, Progress on Drinking-Water and Sanitation: 2012 Update, 2012

[4] Li H, Wan L, Chu G, Tan W, Liu B, Qin Y, Feng Y, Sun D, Fang Y (2017). (Liquid + liquid) extraction of phenols from aqueous solutions with cineole. J Chem Thermodynam.

[5] Sarafraz-Yazdi A, Amiri A (2010). Liquid-phase microextraction. Trends Anal Chem.

[6] Liu X, Ji Y, Zhang Y, Zhang H, Liu M (2007). Oxidized multiwalled carbon nanotubes as a novel solid-phase microextraction fiber for determination of phenols in aqueous samples. J Chromatogr A.

[7] Li QL, Wang XF, Yuan DX (2009). Preparation of solid-phase microextraction fiber coated with single-walled carbon nanotubes by electrophoretic deposition and its application in extracting phenols from aqueous samples. J Chromatogr A.

[8] Zhou FR, Li X, Zeng ZR (2005). Determination of phenolic compounds in wastewater samples using a novel fiber by solid-phase microextraction coupled to gas chromatography. Anal Chim Acta.

[9] Abolghasemi MM, Yousefia V, Amirshaghaghi A (2015). Preparation and evaluation of a layered double hydroxide film on a nanoporous anodic aluminum oxide/aluminum wire as a highly thermal-resistant solid-phase microextraction fiber. New J Chem.

[10] Korba K, Pelit L, Pelit FO, Özdokur KV, Ertas H, Eroglu AE, Ertas F (2013). Preparation and characterization of sodium dodecyl sulfate doped polypyrrole solid phase micro extraction fiber and its application to endocrine disruptor pesticide analysis. J Chromatogr B.

[11] Xiao Z, Zhou X, Niu Y, Yu D, Zhu J, Zhu G (2015). Optimization and application of headspace-solid-phase micro-extraction coupled with gas chromatography–mass spectrometry for the determination of volatile compounds in cherry wines. J Chromatogr B.

[12] Spietelun A, Kloskowski A, Chrzanowsk W, Namieśnik J (2013). Understanding solid-phase microextraction: key factors influencing the extraction process and trends in improving the technique. Chem Rev.

[13] Gonzalez-Toledo E, Prat MD, Alpendurada MF (2001). Solid-phase microextraction coupled to liquid chromatography for the analysis of phenolic compounds in water. J Chromatogr A.

[14] Peñalver A, Pocurull E, Borrull F, Marcé RM (2002). Solid-phase microextraction coupled to high-performance liquid chromatography to determine phenolic compounds in water samples. J Chromatogr A.

[15] Quintana JB, Rodil R, Muniategui-Lorenzo S, Lopez-Mahia P, Prada-Rodriguez D (2007). Multiresidue analysis of acidic and polar organic contaminants in water samples by stir-bar sorptive extraction–liquid desorption–gas chromatography–mass spectrometry. J Chromatogr A.

[16] Montero L, Conradi S, Weiss H, Popp P (2005). Determination of phenols in lake and ground water samples by stir bar sorptive extraction–thermal desorption–gas chromatography–mass spectrometry. J Chromatogr A.

[17] Yu Y, Liu H, Dai X, Cai H, Li C, Yu H (2010). Trace analysis of phenols and chlorophenols in water by in situ derivatization headspace solid-phase microextraction coupled with gas chromatography/mass spectrometry. Chin J Anal Chem.

[18] Haberhauer-Troyer C, Crnoja M, Rosenberg E, Grasserbauer M, Fresenius J (2000). Surface characterization of commercial fibers for solid-phase microextraction and related problems in their application. Anal Chem.

[19] Hu X, Hu Y, Li G (2007). Development of novel molecularly imprinted solid-phase microextraction fiber and its application for the determination of triazines in complicated samples coupled with high-performance liquid chromatography. J Chromatogr A.

[20] Abolghasemi MM, Karimi B, Yousefi V, Behzadnia H, Barzegar H, Piryaei M (2015). Ionic liquid-derived nano-fibrillated mesoporous carbon based on solid-phase microextraction fiber for the analysis of volatile organic compounds from aqueous solutions. New J Chem.

[21] Amanzadeh H, Yamini Y, Masoomi MY, Morsali A (2017). Nanostructured metal–organic frameworks, TMU-4, TMU-5, and TMU-6, as novel adsorbents for solid phase microextraction of polycyclic aromatic hydrocarbons. New J Chem.

[22] Kueseng P, Pawliszyn J (2013). Carboxylated multiwalled carbon nanotubes/polydimethylsiloxane, a new coating for 96-blade solid-phase microextraction for determination of phenolic compounds in water. J Chromatogr A.

[23] Gong SX, Wang X, Chen Y, Cheng CG, Wang ML, Zhao RS (2015). Carboxylated solid carbon spheres as a novel solid-phase microextraction coating for sensitive determination of phenols in environmental water samples. J Chromatogr A.

[24] Wang FX, Zheng J, Qiu JL, Liu SQ, Chen GS, Tong YX, Zhu F, Ouyang GF (2017). In situ hydrothermally grown TiO2@C core-shell nanowire coating for highly sensitive solid phase microextraction of polycyclic aromatic hydrocarbons. Appl Mater Interfaces.

[25] Wang JX, Jiang DQ, Gu ZY, Yan XP (2006). Multiwalled carbon nanotubes coated fibers for solid-phase microextraction of polybrominated diphenyl ethers in water and milk samples before gas chromatography with electron-capture detection. J Chromatogr A.

[26] Kong J, Franklin NR, Zhou C, Chapline MG, Peng S, Cho K, Dai H (2000). Nanotube molecular wires as chemical sensors. Science.

[27] Liu C, Fan YY, Liu M, Cong HT, Cheng HM, Dresselhaus MS (1999). Hydrogen storage in single-walled carbon nanotubes at room temperature. Science.

[28] Iijima S (1991). Helical microtubules of graphitic carbon. Nature.

[29] Iijima S, Ichihashi T (1993). Single-shell carbon nanotubes of 1-nm diameter. Nature.

[30] Basheer C, Alnedhary AA, MadhavaRao BS, Valliyaveettil S, Lee HK (2006). Development and application of porous membrane-protected carbon nanotube micro-solid-phase extraction combined with gas chromatography/mass spectrometry. Anal Chem.

[31] Abolghasemi MM, Yousefi V, Piryaei M (2015). Synthesis of carbon nanotube/layered double hydroxide nanocomposite as a novel fiber coating for the headspace solid-phase microextraction of phenols from water samples. J Sep Sci.

[32] Rastkari N, Ahmadkhaniha R, Yunesian M (2009). Single-walled carbon nanotubes as an effective adsorbent in solid-phase microextraction of low level methyl tert-butyl ether, ethyl tert-butyl ether and methyl tert-amyl ether from human urine. J Chromatogr B.

[33] Ma JP, Xiao RH, Li JH, Yu JB, Zhang YQ, Chen LX (2010). Determination of 16 polycyclic aromatic hydrocarbons in environmental water samples by solid-phase extraction using multi-walled carbon nanotubes as adsorbent coupled with gas chromatography–mass spectrometry. J Chromatogr A.

[34] Cai Y, Jiang G, Liu J, Zhou Q (2003). Multi-walled carbon nanotubes packed cartridge for the solid-phase extraction of several phthalate esters from water samples and their determination by high performance liquid chromatography. Anal Chim Acta.

[35] Li QL, Yuan DX, Lin QM (2004). Evaluation of multi-walled carbon nanotubes as an adsorbent for trapping volatile organic compounds from environmental samples. J Chromatogr A.

[36] Wang X, Li XJ, Li Z, Zhang YD, Bai Y, Liu HW (2014). Online coupling of in-tube solid-phase microextraction with direct analysis in real time mass spectrometry for rapid determination of triazine herbicides in water using carbon-nanotubes-incorporated polymer monolith. Anal Chem.

[37] Cai Y, Jiang G, Liu J, Zhou Q (2003). Multiwalled carbon nanotubes as a solid-phase extraction adsorbent for the determination of bisphenol A, 4-*n*-nonylphenol, and 4-*tert*-octylphenol. Anal Chem.

[38] Cong Hu, Chen Beibei, He Man, Bin Hu (2013). Amino modified multi-walled carbon nanotubes/polydimethylsiloxane coated stir bar sorptive extraction coupled to high performance liquid chromatography-ultraviolet detection for the determination of phenols in environmental samples. J Chromatogr A.

[39] Ai Y, Wu M, Li L, Zhao F, Zeng B (2016). Highly selective and effective solid phase microextraction of benzoic acid esters using ionic liquid functionalized multiwalled carbon nanotubes-doped polyaniline coating. J Chromatogr A.

[40] Geng Y, Liu MY, Li J, Shi XM, Kim JK (1876). Effects of surfactant treatment on mechanical and electrical properties of CNT/epoxy nanocomposites. Compos Part A: Appl Sci Manufac.

[41] Pandey P, Mohanty S, Nayak SK (2014). Tailoring dispersion and interaction of MWNT in polymer nanocomposites, using Triton X-100 as nonionic surfactant. J Mater Eng Perform.

[42] Grossiord N, Loos J, Regev O, Koning CE (2006). Toolbox for dispersing nanotubes into polymers to get conductive nanocomposites. Chem Mater.

[43] Yang K, Wu W, Jing Q, Zhu L (2008). Aqueous adsorption of aniline, phenol, and their substitutes by multi-walled carbon nanotubes. Environ Sci Technol.

[44] Pacheco-Fernandez I, Najafi A, Pino V, Anderson JL, Ayala JH, Afonso AM (2016). Utilization of highly robust and selective crosslinked polymeric ionic liquid-based sorbent coatings in direct-immersion solid-phase microextraction and high-performance liquid chromatography for determining polar organic pollutants in waters. Talanta.

[45] Vaisman L, Marom G, Wagner HD (2006). Dispersions of surface-modified carbon nanotubes in-soluble and water-insoluble polymers. Adv Funct Mater.

[46] Tanimu A, Jillani SMS, Alluhaidan AA, Ganiyu SA, Alhooshani K (2019). 4-phenyl-1,2,3-triazole functionalized mesoporous silica SBA-15 as sorbent in an efficient stir bar-supported micro-solid-phase extraction strategy for highly to moderately polar phenols. Talanta.

[47] Seebunrueng K, Dejchaiwatana C, Santaladchaiyakit Y, Srijaranai S (2017). Development of supramolecular solvent based microextraction prior to high performance liquid chromatography for simultaneous determination of phenols in environmental water. RSC Adv..

