# Supplementary material for: A simple strategy based on fibers coated with surfactant-functionalized multiwalled carbon nanotubes to improve the properties of solid-phase microextraction of phenols in aqueous solution
Source: BMC Chem. 2020 Feb 19;14(1):15. doi: 10.1186/s13065-020-00665-7 (PMC7029595; doi:10.1186/s13065-020-00665-7)
Supplement: Supplementary file 1 — Additional file 1. Additional tables. [file 13065_2020_665_MOESM1_ESM.docx]

**A simple strategy based on fibers coated with surfactant-functionalized multiwalled carbon nanotubes to improve the properties of solid-phase microextraction of phenols in aqueous solution**

**Additional material**

**Experimental method**

Surfactant functionalized MWCNTs

Firstly, 2 g of pristine multi-walled carbon nanotubes powder was added in a 100mL mixture of HNO_3_ and H_2_SO_4_ (V/V = 1:3) in a water bath at 80 °C for 6h with stirring. The processed MWCNTs were gathered by filtration and washed with deionized water until the final pH approached neutral. Secondly, the processed MWCNTs were modified by sodium deoxycholic acid (NaDC) with a mass ratio of 1:1. Table S1 shows the structure and manufacturer of NaDC. MWCNTs/NaDC suspension was prepared according to table S2, and the suspension was ultrasonic for 30 min in the ultrasonic crushing instrument, and then by magnetic stirring for 12h. Finally, the above solution was centrifuged at a centrifuge of 7500 RPM for 30 min, and the precipitation was collected and dried for 24 hours at 60°C to obtain the MWCNTs/NaDC powder.

Table S1 The structure and manufacturer of NaDC

| Surfactant | Structure | content | manufacturer |
| --- | --- | --- | --- |
| NaDC | 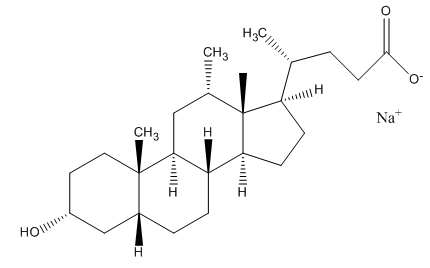 | 99% | Aladdin Chemistry |

Table S2 Preparation of the MWCNTs/NaDC solution

| Surfactant | Surfactant type | CMC | Surfactant weight | MWCNTs weight | Water volume |
| --- | --- | --- | --- | --- | --- |
| NaDC | Anionic | 6×10^-3^ | 2g | 2g | 10mL |

CMC is the critical micelle concentration of surfactant.
